# Supplementary figures and images for: Signaling Peptide SpoV Is Essential for Streptococcus pyogenes Virulence, and Prophylaxis with Anti-SpoV Decreases Disease Severity
Source: Microorganisms. 2021 Nov 10;9(11):2321. doi: 10.3390/microorganisms9112321 (PMC8619256; doi:10.3390/microorganisms9112321)

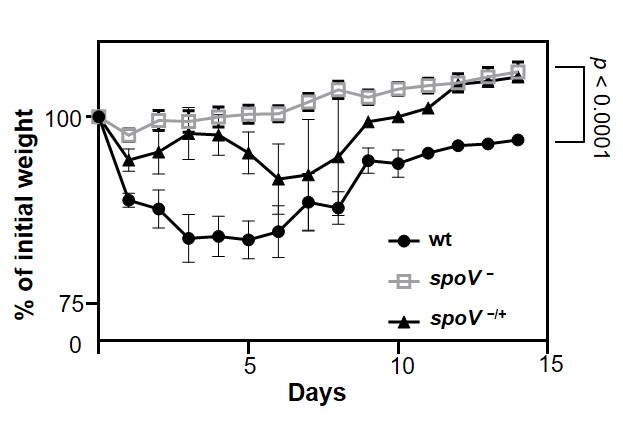

Supplement: Supplementary file 1 [file microorganisms-09-02321-s001.zip › microorganisms-1426515-supplementary.jpg]
